# Root Resorption of Teeth Adjacent to Untreated Impacted Maxillary Canines: A CBCT Study

**DOI:** 10.1155/2021/6635575

**Published:** 2021-04-09

**Authors:** Israa Hussein Ali, Bassam Ali Al-Turaihi, Lamis Khidher Mohammed, Mohammad Khursheed Alam

**Affiliations:** ^1^Department of Pediatric Dentistry, Orthodontics and Preventive Dentistry, College of Dentistry, University of Babylon, Al-Hilla, Babil, Iraq; ^2^Department of Orthodontics, College of Dentistry, University of Al-Kafeel, Al-Najaf, Iraq; ^3^Orthodontics, Department of Preventive Dentistry, College of Dentistry, Jouf University, Sakaka, Aljouf, Saudi Arabia

## Abstract

**Objectives:**

The aim of this retrospective study was to determine the position of the impacted maxillary canine (IMC) and then to inspect the frequency, location, and extent of the consequent root resorption (RR) of the adjacent teeth by using CBCT.

**Materials and Methods:**

Forty-one patients aged 12-68 who have 56 IMC detected on CBCT images were retrospectively examined in 3D. The canine position was determined by assessing the side of impaction, buccopalatal location, the distance of the canine cusp tip to the midline and to the occlusal plane, and canine angulation to the midline. RR of adjacent teeth was analyzed by investigating the nearest location of ectopic canine to adjacent teeth in horizontal and vertical dimensions in addition to measuring the degree of RR.

**Results:**

Twenty-seven (48.2%) impacted canines were on the right side, and 29 (51.8%) were on the left. Most of these teeth 31 (55.4%) were located palatally, while buccal impaction was seen in only 13 (23.2%) canines and 12 (21.4%) were located centrally. The mean distance of the ectopic canine cusp tip to the occlusal plane was significantly higher in males (14.4 mm) than in females (10.7 mm). RR was seen in 9 central incisors (31.03%) and 21 lateral incisors (41.17%) as well as one case in the first and second premolar. This RR was slight for all the adjacent central incisors and premolars.

**Conclusions:**

IMC is more frequent in females, palatally and apically. When left untreated, it may cause RR of some of the adjacent teeth with various degrees, however, with no gender preference.

## 1. Introduction

Permanent tooth impaction usually results from failure of the tooth to emerge into its normal position in the dental arch within the usual period of growth [1–3]. Maxillary canine (MC) is the second most common tooth to be impacted following third molars [2, 4, 5]. The prevalence of IMC ranged from 1% to 3% depending on the population studied [1, 2, 6, 7].

The most frequent complication correlated to IMC is RR of the nearby lateral incisor [3, 6, 8]. When the ectopic canine is situated more mesially, the central incisor is sometimes involved as well. Although uncommon, premolar resorption has also been reported in the literature [1, 6]. Due to absence of symptoms, RR is almost impossible to be diagnosed clinically [8]. Even when the pulp is exposed, this complication remains asymptomatic, resulting in delaying its diagnosis until clinically evident which may ultimately require extraction of the affected tooth [6]. A diverge variation is present in reporting RR of teeth adjacent to IMC [9] ranging from 12.5% [10] in a study that used intraoral radiographs to 66.7% in a three-dimensional CBCT study [11].

The conventional two-dimensional radiograph is the standard primary diagnostic technique used for the localization of IMC, planning treatment, and assessment of treatment outcomes [5, 6]. However, because of the drawbacks of using this technique [1, 12], the clinician may need to use the 3D CBCT to localize the IMC more precisely for minimal invasive surgery and to detect its negative effect on the roots of adjacent teeth more efficiently [6, 13, 14]. This 3D technique can provide overlap-free sagittal, axial, and coronal images for the dental structure in question. It also has an advantage of using significantly less radiation dose compared to the traditional CT scan [1, 15, 16].

As far as we know, no studies were carried out in Iraq to examine the effect of IMC on adjacent teeth and more specifically RR. Therefore, the aim of this retrospective study was to firstly assess the three-dimensional location of IMC in a sample of Iraqi population and secondly to investigate the frequency, location, and extent of RR of adjacent teeth by using CBCT.

## 2. Materials and Methods

Over a period of two years (from July 2018 to August 2020), the CBCT of all patients referred for diagnostic and orthodontic purposes at a private clinic in Al-Hilla city, Babil, Iraq, were examined and only the ones with IMC (s) were selected to assess the unfavorable effect of IMC on adjacent teeth.

The inclusion criteria for the involved cases were as follows:

- Patients aged 12 years or older
- Patients had been referred for a CBCT imaging because of ectopic eruption of one or both MC (s) as detected by clinical examination

The exclusion criteria included the following:

- Patients younger than 12 years
- Patients who had previous or ongoing orthodontic treatment
- Patients who presented intraosseous pathosis related to the studied anterior maxilla
- Patients who had systemic disease or significant medical history

A total of 41 CBCTs, 9 of males and 32 of females, aged 12-68 (mean 20.8 years) were retrospectively analyzed. These tomographs had 56 impacted canines, 26 of them were unilaterally IMC, and 15 had bilateral IMC (Table 1). The chosen MC (s) were considered to be impacted when the MC on the opposite side was fully erupted completely in unilateral cases or when the root formation was complete in bilateral cases [17].

## 3. Radiographic Assessment

The patients were scanned while in a standing position using a CS 9300 3D (Carestream Dental LLC., Atlanta, USA) with 8 × 8 cm field of view (FOV). The exposure parameters were 80 kVp, 10 mA, and 20 s. The CBCT images were assessed in all 3D (axial, sagittal, and coronal) on a flat screen monitor by an orthodontist with 6 years of experience using the CS 3D imaging software (Carestream Dental LLC., Atlanta, USA).

**Figure 1:**
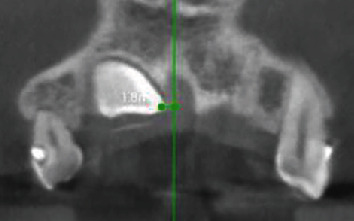
Transverse distance of impacted canine to the midline.

**Figure 2:**
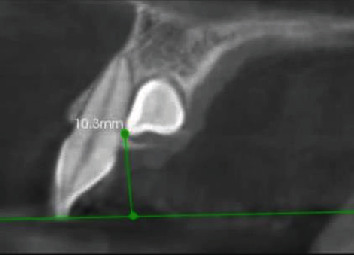
Vertical distance of impacted canine to the occlusal plane.

**Figure 3:**
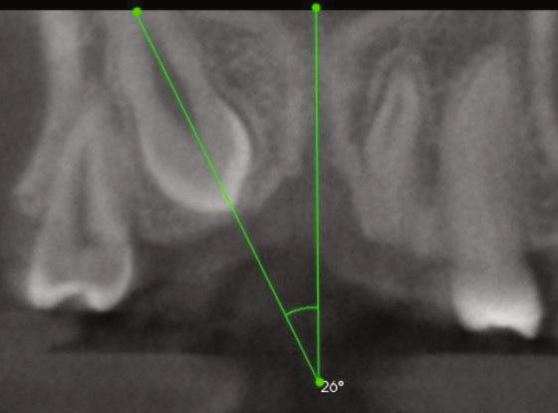
Angulation of the impacted canine to the midline.

**Figure 4:**
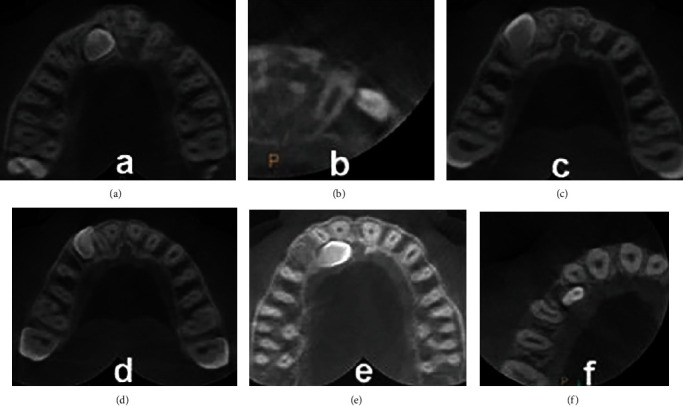
Horizontal location of the impacted canine to the adjacent teeth: (a) apical; (b) distal; (c) buccal; (d) distobuccal; (e) palatal; (f) distopalatal.

**Figure 5:**
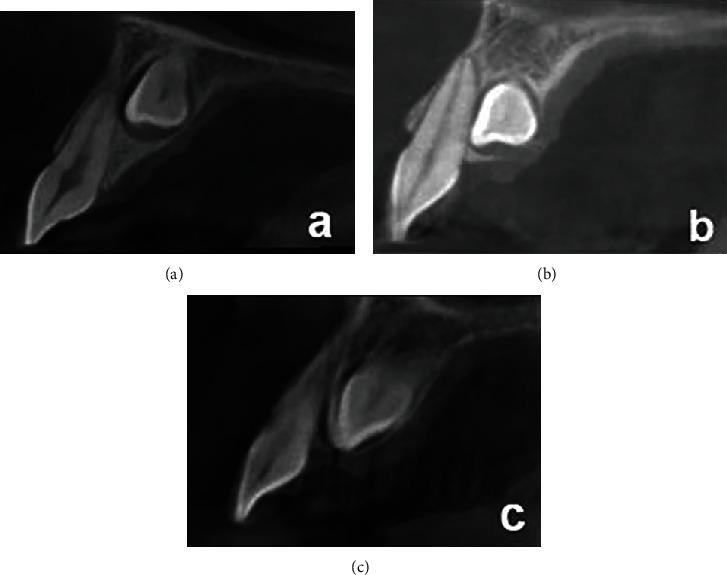
Vertical location of the impacted canine to the adjacent teeth: (a) apical; (b) middle; (c) coronal.

**Figure 6:**
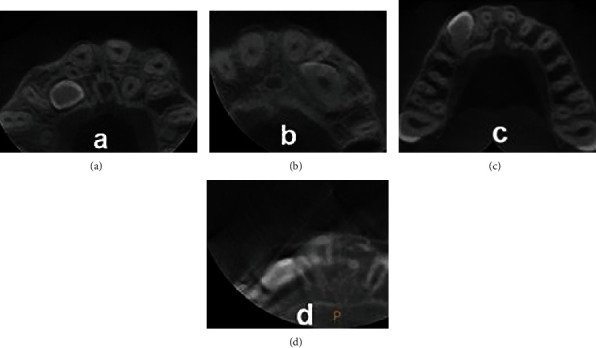
Degree of root resorption of the teeth adjacent to impacted canine: (a) no resorption; (b) slight resorption; (c) moderate resorption; (d) severe resorption.

Radiographic variables of the fifty-six IMC (s) were examined and documented involving the following:

- The 3D localization of the IMC include the following:

1. Side of impaction (bilateral or unilateral (Right or left))
2. Buccopalatal location of IMC in the axial plane (buccal or palatal)
3. Transverse distance of the IMC cusp tip to the midline in the coronal plane (Figure 1)
4. Vertical distance of the IMC cusp tip to the occlusal plane in the sagittal plane (Figure 2)
5. Angulation of the IMC to the midline in the coronal plane (angle between the maxillary arch midline and the long axis of the IMC) (Figure 3)

(ii) RR of teeth adjacent to the IMC (s) was evaluated by determining these three variables:

1. Nearest location of the IMC to the adjacent teeth in horizontal dimension (apical, distal, buccal, distobuccal, palatal, distopalatal) (Figure 4)
2. Nearest location of the IMC to the adjacent teeth in vertical dimension (apical, middle, coronal thirds of the root) (Figure 5)
3. The extent of RR was assessed by using Ericson and Kurol system [18, 19] with grade 1, no RR (undamaged root surface); grade 2, slight RR (up to one-half of dentin thickness); grade 3, moderate RR (halfway or more to pulp, but pulp covered with unbroken dentin); and grade 4, severe RR with pulp exposure (Figure 6)

## 4. Statistical Analysis

Relations of the variables were tested by using chi-square test with Fisher's exact test, whereas variations in means between two groups were assessed by using independent sample *t*-test using SPSS (version 23) (SPSS INC., Chicago, Illinois, USA).

Ten consecutive cases were reevaluated by a second experienced dentist to assess the interrater agreement. Kappa statistics (Cohen's kappa) was used to measure level of agreement for the categorical variables, and intraclass correlation (ICC) was used for the continuous variables.

## 5. Results

Bilateral IMC was found in 15 (3 male, 12 female) patients, and unilateral IMC was detected in 26 (6 male, 20 female) patients (Table 1). In total, twenty-seven (48.2%) IMC (s) were positioned on the right side and 29 (51.8%) were positioned on the left side with no statistically significant difference between males and females. Regarding IMC buccopalatal location, most of the IMC 31 (55.4%) were located palatally, while buccal IMC was seen in only 13 (23.2%) MC (s) and 12 (21.4%) canines were located within the maxillary arch. Despite being located more frequently palatally, no significant difference was found between males and females (*p* < 0.05) (Table 2).

Statistically insignificant difference was found for the distance of the IMC to the midline or IMC angulation (*p* > 0.05). IMC cusp tip to the occlusal plane was significantly higher in males than in females (*p* < 0.05) (Table 3). Statistically insignificant difference was found for the means of the distance of the IMC cusp tip to the midline and to the occlusal plane or canine's angulation to the midline between the right and left sides (*p* > 0.05) (Table 4).

Most of the IMC (s) 51 (25 on the right and 26 on the left) approach the roots of the adjacent maxillary lateral incisors, whereas twenty-nine (14 on the right and 15 on the left) IMC (s) reach the roots of maxillary central incisors. Only one IMC was positioned ectopically posteriorly and was near the roots of the adjacent first and second premolars (Table 5). More than half of the IMC (s) were located in palatal or distopalatal positions in relation to both central and lateral incisors (*p* > 0.05).

Approximately half of the IMC (s) adjacent to the central and lateral incisors were located at the apical third, whereas the IMC close to the first and second premolars was located at the cervical third of their roots (Table 6). Significant difference was found for the lateral incisors between males and females (*p* < 0.05).

RR is detailed in Table 7. Out of the 25 IMC (s) that were approaching both of the adjacent central and lateral incisors, only three canines caused slight RR of both central and lateral incisors. Similarly, for the 15 bilateral cases, most of them had RR on either side, and only four cases show RR on both sides. Insignificant gender disparities were observed (*p* < 0.05).

The interrater agreement was excellent for all continuous variables as well as the buccopalatal location of the IMC and horizontal location of RR, whereas the agreement was substantial for the vertical location of RR and for the degree of RR (Table 8).

## 6. Discussion

Orthodontic intervention to manage IMC (s) varies depending on its position within the maxillary arch in all three dimensions as well as its impact on the neighboring teeth, mainly whether it is causing root resorption of the adjacent teeth and if it did, to what extent that resorption can take place [17]. Therefore, the objectives of this study were to reliably assess these variables in an Iraqi sample by using the accurate diagnostic potency of the CBCT [3, 6, 15]. IMC (s) were located mostly palatally within the arch and in close proximity to the apical third of the adjacent incisors, and root resorption was detected on 31.03% and 41.17% of adjacent central and lateral incisors with the majority being slight degree.

The present study involved scanning and analyzing the CBCT images of patients who have their upper anterior region scanned for diagnostic and orthodontic purposes. Therefore, the results of this study could be considered as a representative sample of patients who have IMC (s). The age group selected to be examined started from 12 years. This is because radiographic investigation of the MC is not required prior to ten years of age and potential IMC can only be seen for older age groups [8, 10].

For gender distribution, the majority of the CBCTs were for females (78%) that were similar to the percentage reported in previous studies [2, 15, 17, 20]. It was also consistent with what has been documented in the literature as a greater prevalence of IMC in females than in males [1, 4, 21]. Such difference could be attributed to the variations in genetics or overall growth and development of craniofacial structures between the two genders [11]. Another possible reason could be that females may pursue orthodontic treatment more frequently than males [2, 17]. For the side of impaction, bilateral cases represent 36.6% of the collected sample which was identical to a previous study [22].

Regarding buccopalatal location of the IMC, preceding studies [2, 15, 17] have found that IMC is displaced more frequently palatally than buccally. The findings of our study were in agreement with these reports as (55.4%) of the inspected IMC were positioned palatally. On the other hand, 23.2% of the IMC in our study were positioned buccally which coincides with a prior study [10]. Similarly, IMC (s) that were in line within the arch were seen in 21.4% in the present study; this was comparable to former studies [23, 24].

For the location of the ectopic canine, the mean distance of its cusps tip to the occlusal plane was 11.5 mm which was slightly higher than the values previously reported, [17, 25] while the mean distance to the midline was 7.8 mm which was lower than the values registered priorly [17]. Such difference could be caused by variation in the groups studied. Regarding canine angulation to the midline, the mean value for this variable was found to be 35.9° which was similar to an earlier study, [22] but higher than the other two [17, 25].

On the other hand, the mean vertical position of the impacted canine in relation to the occlusal plane was significantly higher in males (14.4 mm) than in females (10.7 mm). The reason for this difference is probably because males tend to have significantly larger dentoalveolar heights than females [26] making the IMC located relatively farther from the occlusal plane in males compared to females.

Most of the IMC (s) (almost half of them) were in close proximity to the apical thirds of adjacent central and lateral incisors, whereas 27.6% of centrals and 33.3% of lateral incisors were at the middle thirds; 20.7% of centrals, 17.6% of laterals, and the only first and second premolars were at the cervical thirds which coincides with a preceding study [18].

A wide divergence is present in reporting the prevalence of RR of teeth. This could be due to difference in the radiographic technique or imaging machine used, or it could be a result of variation in the population studied [8]. As the two-dimensional periapical and panoramic radiography can only detect lateral and apical RR and cannot show the buccal and palatal resorption, fifty percent of RR of the adjacent teeth can be overlooked [1, 2, 4, 17]. Therefore, by using the 3D techniques, the diagnostic accuracy and percentage of RR can be increased significantly because these techniques provide information in 3D of the skull [3, 6, 15, 27]. Subsequently, the treatment plan for managing IMC may be changed [24, 28].

Regarding the extent of RR, 69% of adjacent central incisors and 59% of adjacent lateral incisors had no RR. This leaves 31.03% of the neighboring central incisors and 41.17% of the adjacent lateral incisors as well as the first and second premolars having some sort of RR. The percentage for the lateral incisor RR was comparable to previous studies [18, 25]. However, it was lower than other studies [11, 24] and higher than another [2, 29]. Similarly, the percentage for the central incisor RR in this study was higher than former studies [2, 11, 18, 29]. For the premolars, only 1.8% of the examined cases showed RR for the first and second premolars which was lower than earlier studies [2, 29]. For bilateral IMC (s), the results of our study showed no case with RR of all four central incisors which agrees with past studies [20, 23].

Only three IMC (s) (5.36%) caused slight RR of both of the adjacent central and lateral incisors. This finding was similar to Ericson and Kurol's (2000) study [18] who found that only seven (4.49%) out of 156 canines caused RR of both central and lateral incisors. Also, this study has found that there was no gender difference present for the severity of RR which also coincided with previous studies [2, 20].

None of the adjacent central incisors and only one case (1.96%) of adjacent lateral incisors had severe RR, (3.9%) had moderate RR, whereas 35.3% had slight RR which was similar to a former study [15]. This finding disagrees with Ericson and Kurol (1987) [10] who found that half of the resorbed lateral incisors and one-sixth of the resorbed central incisors showed severe RR. Later, Ericson and Kurol (2000) [18] have reported an even higher percentage of severe RR in their CT study with 60% of the lateral incisors and 43% of the central incisors suffering RR that exposes the pulp. Similarly, Rimes et al. (1997) [23] have found that most of the adjacent incisors (85.7%) had severe RR, whereas Dağsuyu et al. (2018) [17] have found that only 6% (10 lateral incisors and four first premolars) of their sample had severe RR.

The orthodontist and a pediatric dentist interrater agreement was consistent with the reliability reported previously [2, 22, 25]. Although the interrater reliability was high in our study, more studies are required to assess whether the diagnostic parameters can be refined to achieve a higher reliability score.

## 7. Conclusion

This study has derived the following conclusion with high interrater correlation:

1. Ectopic eruption of MC occurs more frequently in females than in males by a 3.5-fold margin
2. Palatal IMC was nearly two and a half times higher than buccal impaction
3. Most of the IMC (s) were situated apically (almost half of the assessed teeth)
4. No significant difference was found between males and females for the three-dimensional position of the IMC apart from the vertical distance of the MC cusp tip to the occlusal plane
5. RR affected approximately one-third of the adjacent teeth in untreated patients who have IMC
6. When present, RR mostly involved adjacent lateral incisors (41%), followed by central incisors (31%), and the only case of first and second premolars (1.8%)
7. The degree of RR was predominantly slight for all the affected neighboring teeth
8. Gender was not significantly related to the RR of the neighboring teeth

## Figures and Tables

**Table 1:** Distribution of impacted maxillary canines.

		Age (years)		Impacted maxillary canines		
	No.	Range	Mean ± SD	Unilateral	Bilateral	Total
Males	9	12-68	21.8 ± 17.6	6	3	12
Females	32	12-45	20.5 ± 8.9	20	12	44
Total	41	12-68	20.8 ± 11.1	26	15	56

**Table 2:** Side and buccopalatal location of impacted maxillary canines according to gender.

Gender	Right side	Left side	Total	Buccally impacted	Palatally impacted	Centrally impacted	Total
Male	6 (50%)	6 (50%)	12 (100%)	4 (33.3%)	6 (50%)	2 (16.7%)	12 (100%)
Female	21 (47.7%)	23 (52.3%)	44 (100%)	9 (20.5%)	25 (56.8%)	10 (22.7%)	44 (100%)
Total	27 (48.2%)	29 (51.8%)	56 (100%)	13 (23.2%)	31 (55.4%)	12 (21.4%)	56 (100%)

**Table 3:** Comparisons of impacted maxillary canine cusp tip distance to the midline and to the occlusal plane and canine's angulation to the midline for male and female subjects.

Variables	Total (*n* = 56)	Males (*n* = 12)	Females (*n* = 44)	*p* value
	Mean ± SD	Mean ± SD	Mean ± SD	
Distance of impacted canine cusp tip to the midline	7.82 ± 5.35	8.28 ± 5.97	7.69 ± 5.24	0.742
Distance of impacted canine cusp tip to the occlusal plane	11.52 ± 4.71	14.42 ± 6.14	10.73 ± 3.97	0.015
Angulation of impacted canine to the midline	35.92 ± 21.85	34.19 ± 15.42	36.40 ± 23.43	0.760

^∗^ *p* < 0.05, significant difference.

**Table 4:** Comparisons of right and left impacted maxillary canine cusp tip distance to the midline and to the occlusal plane as well as canine's angulation to the midline.

Variables	Right side (*n* = 27)	Left side (*n* = 29)	*p* value
	Mean ± SD	Mean ± SD	
Distance of impacted canine cusp tip to the midline	8.35 ± 5.99	7.32 ± 4.73	0.479
Distance of impacted canine cusp tip to the occlusal plane	11.44 ± 4.94	11.59 ± 4.57	0.909
Angulation of impacted canine to the midline	30.41 ± 23.72	41.06 ± 18.95	0.068

^∗^ *p* < 0.05, significant difference.

**Table 5:** Location of impacted canine in relation to the adjacent teeth in a horizontal dimension.

Location of impacted canine in horizontal dimension	Apical	Distal	Palatal	Distopalatal	Buccal	Distobuccal	Total
Central incisor	5	1	7	15	—	1	29
Lateral incisor	11	1	13	14	2	10	51
First premolar	—	—	—	—	—	1	1
Second premolar	—	—	—	—	1	—	1

**Table 6:** Location of impacted canine in relation to the adjacent teeth in a vertical dimension.

Location of impacted canine in vertical dimension	Apical third	Middle third	Cervical third	Total
Central incisor	15 (51.7%)	8 (27.6%)	6 (20.7%)	29
Lateral incisor	25 (49.02%)	17 (33.3%)	9 (17.65%)	51
First premolar	—	—	1 (100%)	1
Second premolar	—	—	1 (100%)	1

**Table 7:** Degree of root resorption for the impacted canines in relation to the adjacent teeth.

Degree of root resorption on adjacent teeth	No resorption	Slight resorption	Moderate resorption	Severe resorption	Total
Central incisor	20	9	—	—	29
Lateral incisor	30	18	2	1	51
First premolar	—	1	—	—	1
Second premolar	—	1	—	—	1

**Table 8:** Interrater agreement.

Variable	Kappa test	Intraclass correlation
Buccopalatal location of impacted canine	1	
Horizontal location of root resorption	0.865	
Vertical location of root resorption	0.667	
Severity of root resorption	0.697	
Angulation of impacted canine		0.987
Distance of impacted canine to the midline		0.980
Distance of impacted canine to the occlusal plane		0.960

## Data Availability

Details are presented within the article in the form of tables and text in results. Other data will be made available upon request.
